# Disclosure of a Promising Lead to Tackle Complicated Skin and Skin Structure Infections: Antimicrobial and Antibiofilm Actions of Peptide PP4-3.1

**DOI:** 10.3390/pharmaceutics13111962

**Published:** 2021-11-19

**Authors:** Ana Gomes, Lucinda J. Bessa, Iva Fernandes, Ricardo Ferraz, Cláudia Monteiro, M. Cristina L. Martins, Nuno Mateus, Paula Gameiro, Cátia Teixeira, Paula Gomes

**Affiliations:** 1 LAQV-REQUIMTE, Departamento de Química e Bioquímica, Faculdade de Ciências, Universidade do Porto, P-4169-007 Porto, Portugal; anagomes@fc.up.pt (A.G.); lbessa@egasmoniz.edu.pt (L.J.B.); iva.fernandes@fc.up.pt (I.F.); ricardoferraz@eu.ipp.pt (R.F.); nbmateus@fc.up.pt (N.M.); agsantos@fc.up.pt (P.G.); catia.teixeira@fc.up.pt (C.T.); 2 Centro de Investigação Interdisciplinar Egas Moniz (CiiEM), Egas Moniz-Cooperativa de Ensino Superior, CRL, P-2829-511 Almada, Portugal; 3 Ciências Químicas e das Biomoléculas–CISA, Escola Superior de Saúde, Politécnico do Porto, P-4200-072 Porto, Portugal; 4 i3S-Instituto de Investigação e Inovação em Saúde, Universidade do Porto, P-4200-135 Porto, Portugal; claudia.monteiro@ineb.up.pt (C.M.); cmartins@ineb.up.pt (M.C.L.M.); 5 INEB-Instituto de Engenharia Biomédica, P-4200-135 Porto, Portugal; 6 ICBAS, Instituto de Ciências Biomédicas Abel Salazar, Universidade do Porto, P-4050-313 Porto, Portugal

**Keywords:** antibacterial, antibiofilm, antifungal, antimicrobial peptides, collagen, multidrug resistance, skin infections, wound healing

## Abstract

Efficient antibiotics are being exhausted, which compromises the treatment of infections, including complicated skin and skin structure infections (cSSTI) often associated with multidrug resistant (MDR) bacteria, methicillin-resistant *S. aureus* (MRSA) being the most prevalent. Antimicrobial peptides (AMP) are being increasingly regarded as the new hope for the post-antibiotic era. Thus, future management of cSSTI may include use of peptides that, on the one hand, behave as AMP and, on the other, are able to promote fast and correct skin rebuilding. As such, we combined the well-known cosmeceutical pentapeptide-4 (PP4), devoid of antimicrobial action but possessing collagenesis-boosting properties, with the AMP 3.1, to afford the chimeric peptide PP4-3.1. We further produced its *N*-methyl imidazole derivative, MeIm-PP4-3.1. Both peptide-based constructs were evaluated in vitro against Gram-negative bacteria, Gram-positive bacteria, and *Candida* spp. fungi. Additionally, the antibiofilm activity, the toxicity to human keratinocytes, and the activity against *S. aureus* in simulated wound fluid (SWF) were assessed. The chimeric peptide PP4-3.1 stood out for its potent activity against Gram-positive and Gram-negative bacteria, including against MDR clinical isolates (0.8 ≤ MIC ≤ 5.7 µM), both in planktonic form and in biofilm matrix. The peptide was also active against three clinically relevant species of *Candida* fungi, with an overall performance superior to that of fluconazole. Altogether, data reveal that PP4-3.1 is as a promising lead for the future development of new topical treatments for severe skin infections.

## 1. Introduction

Complicated skin and skin structure infections (cSSTI) are often caused by multidrug resistant (MDR) pathogens from the so-called ESKAPE (*Enterococcus faecium, Staphylococcus aureus, Klebsiella pneumoniae, Acinetobacter baumannii, Pseudomonas aeruginosa, and Enterobacter species*) group, towards which available antibiotics are on the edge of becoming ineffective [1]. For instance, it is fairly common nowadays to isolate *P. aeruginosa* and *A. baumannii* that are resistant to carbapenems and third generation cephalosporins, which are last-line antibiotics to fight these bacteria. In the last several years, worldwide awareness has been triggered about the menace of antimicrobial resistance. It has been estimated that by 2050 over 10 million deaths will occur due to this appalling problem [2]. Accordingly, the World Health Organization (WHO) published in 2017 a priority list of MDR bacteria for which efficient treatments are urgently needed [3]. In light of this, and within scope of the continuous search for new pathways to fight MDR bacteria, antimicrobial peptides (AMP) have emerged as an attractive alternative for conventional antibiotics [4]. AMP typically display broad-spectrum activity at very low doses and are unlikely to induce bacterial resistance [5]. They can also act synergistically with conventional antibiotics, reducing the required dose of the latter and thereby decreasing their side effects and capability to induce pathogen resistance [6].

Regarding cSSTI treatment, most complicated cases may require wound debridement to remove the bacterial biofilms formed, which contribute not only to the severity and chronicity of infection, but also to the delay or even impairment of the healing process [7]. However, debridement often does not suffice to ensure complete eradication of the biofilms, and a complementary antibiotic treatment is needed. As such, after debridement, there is a “window of opportunity” where topically applied antiseptics could have crucial action in avoiding recurrence of the infection [8,9,10]. In parallel, timely application of adequate topical formulations could further contribute to prepare the wound bed for fast and correct healing. Such dual–antimicrobial plus healing–action may be achieved by embedding antiseptic agents into suitable scaffolds. In this regard, recent approaches towards the treatment of cSSTI have used matrix scaffolds based on collagen, as these play an important role in several steps of the healing process [11]. For instance, during the inflammation phase, increased concentrations of matrix metalloproteinases (MMPs) in the wound bed cause collagen degradation, which is detrimental for fast healing. Hence, when collagen is topically applied or its production is induced in the wound site, it may act as a decoy for the MMPs, thereby shortening the inflammatory phase and accelerating skin rebuilding [12].

With the above in mind, we previously reported a set of hybrid constructs where a collagen boosting peptide (CBP), the well-known “pentapeptide-4” (PP4), was covalently linked to an antimicrobial peptide (AMP), 3.1, using different orientations, spacers, and *N*-terminal modifications, aiming at a dual-action peptide with therapeutic potential to tackle cSSTI [13,14]. This study revealed that the peptide 3.1-PP4 has potent and selective activity against Gram-negative bacteria, including MDR clinical isolates, both in planktonic and biofilm forms [13]. Besides that, this peptide is easily modified at its *N*-terminus via the copper(I)-catalyzed alkyne-azide cycloaddition (CuAAC) reaction to introduce an imidazolium ionic liquid (IL), affording a new construct, MeIm-3.1-PP4, with higher solubility and enzymatic stability towards tyrosinase [14].

Our previous focus on peptide 3.1-PP4 and its MeIm-3.1-PP4 derivative was mainly driven by the higher selectivity of these constructs against MDR Gram-negative bacteria, which are the most concerning health threat due to lack of efficient alternatives to antibiotics [15]. However, the fact that methicillin-resistant *S. aureus* (MRSA), a Gram-positive bacterium, is the most prevalent pathogen in cSSTI cannot be disregarded [16]. Moreover, it is now well established that most cSSTI have polymicrobial etiology, also involving fungal besides bacterial pathogens. Indeed, although fungal colonization of non-healing wounds has been often overlooked [17], a high prevalence of fungal communities in chronic wounds has been previously reported and associated with both healing time and formation of mixed biofilms with bacteria [18]. Common fungal pathogens in cSSTI include *Cladosporidium* spp. and, mainly, *Candida* spp., with *C. albicans* and *C. parapsilosis* as the most prevalent [19]. Hence, anti-*Candida* activity assessment is also of chief importance when exploring new ways to tackle cSSTI.

In view of the above, we focused our efforts on peptide PP4-3.1, which was previously found to have broader spectrum activity than its reversed order isomer, 3.1-PP4 [13]. In this work we explored peptide PP4-3.1, its methyl imidazolium derivative MeIm-PP4-3.1, and the noncovalent mixture of both peptide building blocks, PP4 and 3.1 (PP4:3.1 in equimolar proportion), regarding their in vitro antibacterial activity against reference and MDR bacterial strains, their antibiofilm activity against MDR clinical isolates of *K. pneumoniae* and *S. aureus*, (iii) their cytotoxicity against HaCaT cell line, and their activity against *Candida* spp. The activity of the best performer of the set, peptide PP4-3.1, was further assessed against *S. aureus* in simulated wound fluid (SWF). Altogether, the data obtained indicate that PP4-3.1 is a valuable lead for advancing novel topical formulations to tackle cSSTI.

## 2. Materials and Methods

### 2.1. Peptide Synthesis

Peptides PP4-3.1, 3.1 and PP4 were assembled by solid-phase peptide synthesis (SPPS), using the Fmoc/^t^Bu orthogonal protection scheme [20]. Briefly, the solid support, Fmoc-Rink-amide MBHA resin (100–200 mesh, 0.52 mmol/g-NovaBiochem, Merck KGaA, Darmstadt, Germany), was first swelled with *N,N*-dimethylformamide (DMF, CARLO ERBA, Val-de-Reuil, France) for 30 min at room temperature (r.t.) and then deprotected (removal of the Fmoc group) by reaction with 20% piperidine (Merck, Darmstadt, Germany) in DMF for 20 min at r.t. After washing with DMF (3 × 10 mL) and dichloromethane (DCM, CARLO ERBA, Val-de-Reuil, France) (3 × 10 mL), the *C*-terminal amino acid residue was incorporated through in situ activation and coupling by adding to the resin a mixture of 5 molar equivalents (eq) of the Fmoc-protected amino acid (Fmoc-AA-OH, Bachem, Bubendorf, Switzerland), 5 eq of *O*-(benzotriazol-1-yl)-*N*,*N*,*N*′,*N*′-tetramethyluronium hexafluorophosphate (HBTU, NovaBiochem, Merck KGaA, Darmstadt, Germany) and 10 eq of *N*-ethyl-*N,N*-diisopropylamine (DIEA, VWR, Radnor, PA, USA) in DMF, and allowing the reaction to occur at r.t for 1 h, under stirring. The peptide chain was grown in the C_t_→N_t_ direction by means of this succession of deprotection, washing, and coupling steps, until the full sequence was assembled. For the modified peptide MeIm-PP4-3.1, once the full sequence of PP4-3.1 was fully assembled, azido acetic acid was coupled similarly to the coupling protocol for Fmoc-AA-OH, i.e., using a mixture containing 5 eq of the azido acetic acid (Sigma-Aldrich, St. Louis, MO, USA), 5 eq of HBTU, and 10 eq of DIEA in DMF, under stirring at r.t. for 1 h. After the washing cycle, the CuAAC “click” reaction was performed on-resin, by adding a solution containing propargyl-MeIm (20 mg, 0.1 mmol, 1 eq) previously synthesized as earlier reported by us, ref. [14] 10 eq of 2,6-lutidine (116 µL, Alfa Aesar, Ward Hill, MA, USA), 1 eq of sodium L-ascorbate (19.8 mg, Sigma-Aldrich, St. Louis, MO, USA), and 10 eq DIEA (170 µL) in DMF (3 mL), followed by addition of 1 eq copper(I)-bromide (14.3 mg, Fluka/Honeywell, Charlotte, NC, USA) in 1 mL acetonitrile (ACN, CARLO ERBA, Val-de-Reuil, France). The reaction was allowed to proceed for 24 h at r.t., after which the resin was washed five times with 10 mL of 0.1 M aqueous ethylenediaminetetraacetic acid (EDTA, PanReac AppliChem GmbH, Darmstadt, Germany), DMF (3 × 10 mL), and DCM (3×10 mL). Once all peptide constructs were fully assembled on the solid support, their full deprotection and release was carried out by acidolysis using a TFA-based cocktail containing 95% TFA (VWR, Radnor, PA, USA), 2.5% triisopropyl silane (TIS, Alfa Aesar, Ward Hill, MA, USA) and 2.5% deionized water. The resulting crude products were purified by preparative RP-HPLC using a Hitachi-Merck LaPrep Sigma system (VWR, Radnor, PA, USA) equipped with an LP3104 UV detector and an LP1200 pump and employing an RP-C18 column (250 × 25 mm, 5 µm pore size). Gradient elution using 0.05% aqueous TFA as a solvent A and ACN as solvent B, varied depending on the crude peptide; however, all elutions were completed in 60 min at a flow-rate of 15 mL/min. The chromatographically pure peptide fractions were collected, pooled, and freeze-dried to afford the final products as fluffy white solids. The purity of the final products was confirmed by RP-HPLC analysis, and their MW confirmed by ESI-IT-MS, on a Finnegan Surveyor LCQ DECA XP MAX spectrometer from Thermo Fisher Scientific (Waltham, MA, USA) operating with electrospray ionization and ion trap quadrupole detection. 

### 2.2. Solutions for In Vitro Assays

Stock solutions of the test peptide-based compounds were prepared in distilled water at approximately 10 mg/mL for testing in vitro antibacterial, antibiofilm and antifungal activity. After the assays, the stock solutions were accurately quantitated using a Thermo Scientific™ NanoDrop™ One microvolume UV-Vis Spectrophotometer (Thermo Fisher Scientific, Waltham, MA, USA), and the MIC values were adjusted accordingly. For the cytotoxicity and activity in SWF assays, peptide solutions were accurately quantitated prior to the assays using the same NanoDrop^TM^ system. In either case, the 31 quantitation method was chosen, where an extinction coefficient ε_205_ of 31 mL⋅mg^−1^⋅cm^−1^ is assumed [21].

### 2.3. Bacterial Strains and Culture Conditions

*Escherichia coli* ATCC 25922, *Pseudomonas aeruginosa* ATCC 27853, *Klebsiella pneumoniae* ATCC 13883, *Staphylococcus aureus* ATCC 29213, *Enterococcus faecalis* ATCC 29212, *Staphylococcus epidermidis* ATCC 14990, and *Streptococcus pyogenes* ATCC 19615 were used in this study, as well as MDR clinical isolates of *K. pneumoniae* (KP010), *S. aureus* (SA007), and *P. aeruginosa* (PA004), whose antimicrobial resistance profile is shown in Table S1 (Supporting Information). These bacteria were all grown at 37 °C for 24 h, on Mueller-Hinton (MH) agar (Liofilchem srl, Roseto degli Abruzzi [Te], Italy) from stock cultures at −80 °C. The exception was *S. pyogenes* ATCC 19615, which was grown in Brain Heart Infusion broth (BHI, Liofilchem srl, Italy) supplemented with 1.5% (*w*/*v*) agar and with 5% Defibrinated Sheep Blood (Thermo Fisher Scientific, Waltham, MA, USA) at 37 °C with 5% CO_2_ for 24 h. 

### 2.4. Antibacterial Activity

The MIC values of the test products were determined through the broth microdilution method against the bacteria listed above. Ciprofloxacin (CIP) was included in the assays as reference antibiotic. The medium used for these assays was the cation-adjusted Mueller-Hinton broth (MHB2, Sigma-Aldrich, St. Louis, MI, USA), except for *S. pyogenes*, where the medium was supplemented with lysed horse blood at 2.5–5% (Sigma-Aldrich, St. Louis, MO, USA), according to the CLSI guidelines [22]. The MBC values were determined as previously described [23].

### 2.5. Antibiofilm Activity

#### 2.5.1. Crystal Violet Assay

Antibiofilm activity was assessed as the ability of the test compounds to inhibit biofilm formation by *S. aureus* (SA007) and *K. pneumoniae* (KP010) MDR clinical isolates. The peptide constructs were tested at concentrations corresponding to their MIC, ½ × MIC, and ¼ × MIC in tryptic soy broth (TSB, Liofilchem s.r.l., Roseto degli Abruzzi, Italy), using the crystal violet assay, as previously described [23]. The results are given as absorbance at 595 nm, and representative of two independent experiments performed in triplicate.

#### 2.5.2. Microscopic Visualization of Biofilms

Biofilms of SA007 and KP010 MDR clinical isolates were allowed to grow on 35 mm high µ-Dishes with ibidi polymer coverslips (ibidi GmbH), in TSB and in TSBG (TSB + 1% Glucose), respectively. The test peptide constructs (PP4-3.1 and MeIm-PP4-3.1) were previously added to each respective medium at concentrations equal to at MIC, ½ × MIC and ¼ × MIC. In the control groups, no peptides were added. After 24 h, in the case of SA007 biofilms, or after 48 h in case of KP010 biofilms, at 37 ℃, they were stained using the Live/Dead staining mixture (LIVE/DEAD BacLight Bacterial Viability Kit, Thermo Fisher Scientific, Waltham, MA, USA) as described by Coelho and co-workers [24], and then visualized under a fluorescence microscope (Leica DMI6000 FFW, Leica Microsystems, Carnaxide, Portugal).

### 2.6. Cell Culture Conditions

Immortalized human keratinocytes (HaCaT cell line) were grown as monolayer from passage number 38–48. For routine maintenance, cells were cultured to 25 cm^2^ as a monolayer and maintained in in Dulbecco’s Modified Eagle Medium (DMEM, CLS) supplemented with 10% fetal bovine serum (FBS, Biowest) and 1% of antibiotic/antimycotic solution (100 units/mL of penicillin, 10 mg/mL of streptomycin, and 0.25 mg/mL of amphotericin B, Sigma-Aldrich, St. Louis, MO, USA). Cells were harvested by trypsinization (0.25% (*w*/*v*) trypsin-EDTA_4_Na) once a week, with a split ratio of 1:10. For freezing procedures, cells were resuspended at a concentration of 2 million/mL of freezing medium (complete medium with 10% DMSO) and the sterile cryovials stored in a liquid nitrogen container.

### 2.7. Toxicity to Human Keratinocytes

For viability assays, HaCaT cells were seeded at 4 × 10^4^ cell/mL. The 96-well plates were incubated at 37 °C in a 5% CO_2_ atmosphere, and the cells allowed to grow until confluency was reached. At this point, the test peptides were added to the wells in the 6.3 to 100 µM concentration range in DMEM with 2% FBS and incubated at 37 °C in a 5% CO_2_ atmosphere. After 24 h, cell viability was accessed using the AlamarBlue™ Cell Viability Reagent (Resazurin sodium salt, Sigma-Aldrich, St. Louis, MO, USA). The medium was removed, and 20 µL of the AlamarBlue™ reagent at 0.15 mg/mL were added to 100 µL of Hank’s Balanced Salt Solution (HBSS, Sigma-Aldrich, St. Louis, MO, USA). The plate was incubated for 2 h at 37 °C in a 5% CO_2_ atmosphere, after which fluorescence was read at 560/590 nm in a Flex Station 3 multi-mode microplate reader (Molecular Devices, California, USA). IC_50_ values, indicating the concentration of test peptides causing a 50% growth inhibition, were determined using GraphPad Prism 9.0 software applying the equation log(inhibitor) versus response with variable slope (four parameters), as described in the Supplementary Materials (Figure S9).

### 2.8. Antifungal Activity

Antifungal activity was assessed against three reference strains of *Candida* spp., namely, *C. albicans* ATCC 90028, *C. glabrata* ATCC 90030, and *C. parapsilosis* ATCC 22019. These strains were grown on Sabouraud agar 4% dextrose agar (Sigma-Aldrich, St. Louis, MO, USA) at 37 °C for 24 h. The MIC values were determined through a broth microdilution method in Roswell Park Memorial Institute (RPMI) 1640 medium, supplemented with glucose to a final concentration of 2% (RPMI 2% G), according to the European Committee on Antimicrobial Susceptibility Testing (EUCAST) protocol [25,26,27,28].

### 2.9. Activity in Simulated Wound Fluid

The activity of PP4-3.1 against *S. aureus* (ATCC 29213) in SWF and MHB (Sigma-Aldrich, St. Louis, MO, USA) was determined. To this end, a peptide stock solution was prepared in water at 10 mg/mL and then diluted in 0.02% aqueous acetic acid containing 0.4% of bovine serum albumin (BSA, Sigma-Aldrich, St. Louis, MO, USA) to a final concentration range from 1280 to 1.25 µg/mL. The SWF was prepared containing 50% FBS and 50% peptone water (0.9% NaCl in 0.1% aqueous peptone, Sigma-Aldrich, St. Louis, MO, USA). *S. aureus* was incubated at 10^5^ CFU/mL in either SWF or MHB after growth in MHB solution for 24 h at 37 °C in the presence of the test peptide within the concentration range 1280 to 1.25 µg/mL. After approximately 18 h of incubation at 37 °C, bacterial growth was monitored, and MIC values determined in triplicates from three independent experiments. The MBC values were also accessed by incubating 10 µL of the content of the first three wells where bacterial growth was not observed, in tryptic soy agar (TSA, Sigma-Aldrich, St. Louis, MO, USA) at 37 °C for about 24 h [29].

### 2.10. Statistics and Data Analysis

The results of inhibition of biofilm formation were expressed as mean ± standard deviation, and statistical comparison between the control and treated biofilms were performed using a Student’s *t*-test (*p* < 0.05 was considered statistically significant).

## 3. Results and Discussion

### 3.1. Peptide Synthesis

Peptides PP4-3.1, PP4 and 3.1 were synthesized through solid phase peptide synthesis (SPPS) as earlier described by us [13]. For the synthesis of MeIm-PP4-3.1, the amino acid sequence of peptide PP4-3.1 was first assembled by SPPS followed by stepwise on-resin N-terminal modification through coupling with azido acetic acid to afford the azide-modified peptide and, subsequently, reaction with propargyl-methylimidazole (Pr-MeIm) via CuAAC to produce the desired final construct, following the procedure previously reported for MeIm-3.1-PP4 [14]. After acidolytic cleavage from the resin support using a trifluoroacetic acid (TFA)-based cocktail, all crude peptide constructs were purified by preparative reverse-phase high performance liquid chromatography (RP-HPLC) and the pure fractions collected, pooled, and freeze-dried. The final peptides (Table 1) were obtained in high purity (>95%), as confirmed by analytical RP-HPLC, and their molecular weights (MW) confirmed by electrospray ionization-ion trap mass spectrometry (ESI-IT MS). Chromatographic and spectral traces are given in the Supplementary Materials (Figures S1–S8).

### 3.2. Antibacterial Activity

The minimum inhibitory concentration (MIC) values for the peptides PP4-3.1, MeIm-PP4-3.1, 3.1, PP4 and a noncovalent mixture of the latter two, indicated as PP4:3.1 (1:1) and the antibiotic ciprofloxacin (CIP), were assessed in MHB2 following the Clinical and Laboratory Standards Institute (CLSI) protocol against Gram-negative (*Escherichia coli*, *Pseudomonas aeruginosa*, *Klebsiella pneumoniae*) and Gram-positive (*Staphylococcus aureus*, *Enterococcus faecalis*) bacterial reference strains (ATCC). The best performing peptides, PP4-3.1 and MeIm-PP4-3.1, were additionally tested against *Staphylococcus epidermidis*, since this species integrates the commensal skin microbiota and interferes with biofilm formation by other species such as *S. aureus* [30], and against *Streptococcus pyogenes*, as this is a major cause of monomicrobial necrotizing soft tissue infection [31].

Data in Table 2 show that, as expected, peptide PP4 was devoid of antibacterial activity, while 3.1 displayed broad spectrum activity as previously reported [13]. The hybrid constructs, PP4-3.1 and MeIm-PP4-3.1, exhibited significant activity with MIC values similar to each other against both ATCC bacterial strains and MDR clinical isolates of *P. aeruginosa* (PA004), *S. aureus* (SA007), and *K. pneumoniae* (KP010). Remarkably, while both PP4-3.1 and MeIm-3.1-PP4 were comparable with CIP against susceptible (ATCC) strains of the tested pathogens, their potency against MDR clinical isolates was significantly higher than that of CIP. For instance, peptide PP4-3.1 was 130-fold and 68-fold more active than CIP against *S. aureus* SA007 and *P. aeruginosa* PA004 isolates, respectively. The MBC values were found to match the MIC values, highlighting the bactericidal action of both peptide constructs. The noncovalent equimolar mixture of PP4 and 3.1 (PP4:3.1) was generally less active than the covalent analogue PP4-3.1, being only slightly more active against *E. faecalis* and less active against *P. aeruginosa* and *K. pneumoniae*. Therefore, the noncovalent mixture PP4:3.1 did not offer any advantage over the covalent construct PP4-3.1 regarding antimicrobial activity.

### 3.3. Antibiofilm Activity

#### 3.3.1. Inhibition of Biofilm Formation by the Crystal Violet Assay

The ability of the best performing peptides, PP4-3.1 and MeIm-PP4-3.1, to inhibit bacterial biofilm formation was assessed against *S. aureus* (SA007) and *K. pneumoniae* (KP010) MDR clinical isolates. The noncovalent equimolar mixture PP4:3.1 was also tested in this regard, but only against the KP010 isolate. To this end, biofilms were formed in the presence of the test peptides at MIC and sub-MIC (½ × MIC and ¼ × MIC) and in the absence of peptide (control). Noteworthy, this type of assay was carried out in TSB [32], in which MIC values may differ from those obtained in the antibacterial activity assay carried out in MHB2 and listed in Table 2. Hence, MIC values referred to in this section are those displayed by the test peptides in TSB, namely, 1.3, and 1.4 µM against *S. aureus* (SA007) and 5.7, 10.9 and 15 µM against *K. pneumoniae* (KP010) for PP4-3.1, MeIm-PP4-3.1 and PP4:3.1 respectively.

**Figure 1:**
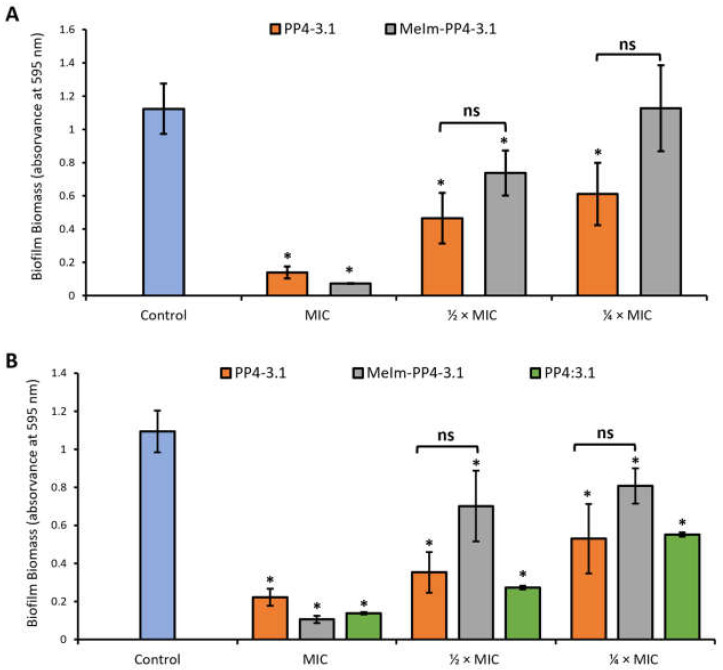
Biofilm biomass formed by MDR clinical isolates of (**A**) *S. aureus* (SA007) and (**B**) *K. pneumoniae* (KP010), in the presence of PP4-3.1, MeIm-PP4-3.1 and the noncovalent equimolar mixture PP4:3.1. The test peptides were added to culture medium (TSB) at MIC, ½ × MIC and ¼ × MIC (with reference to MIC values obtained in TSB, see text). Control biofilms were grown in the absence of peptide. Data are representative of two independent experiments performed in triplicate. The error bars represent the standard deviation (SD). ns: not significant; Statistically significant differences between biofilms formed in the presence of the peptides and respective control biofilms without peptide (*p* < 0.05) are marked with an asterisk (∗).

The biofilm biomass formed in each case was quantified through the crystal violet assay, being expressed as absorbance at 595 nm (Figure 1). As expected, only minimal biofilm formation could be observed in the presence of the test peptides at their MIC for both the Gram-positive and the Gram-negative MDR clinical isolates. In the specific case of the SA007 MDR isolate (Figure 1A), both PP4-3.1 and MeIm-PP4-3.1 were able to inhibit biofilm formation at sub-inhibitory concentrations without significant statistical difference between them. The same behavior was observed against KP010 MDR isolate; however, the MIC of MeIm-PP4-3.1 against this bacterial isolate in TSB (10.9 µM) roughly doubled that of PP4-3.1 (5.7 µM), which means that a much lower concentration of PP4-3.1 was required to exert an antibiofilm action like that of MeIm-PP4-3.1.

The equimolar PP4:3.1 mixture had a similar inhibitory profile to that of its covalent counterpart PP4-3.1 on biofilms of the KP010 isolate. Still, these identical effects were observed for test peptides whose MIC values considerably differed in TSB (Table 3), i.e., a much lower amount of the covalent analogue PP4-3.1 (5.7 µM), as compared to the noncovalent mixture (15 µM), was needed to inhibit biofilm formation by a similar extent.

#### 3.3.2. Inhibition of Biofilm Formation: Microscopic Visualization 

**Figure 2:**
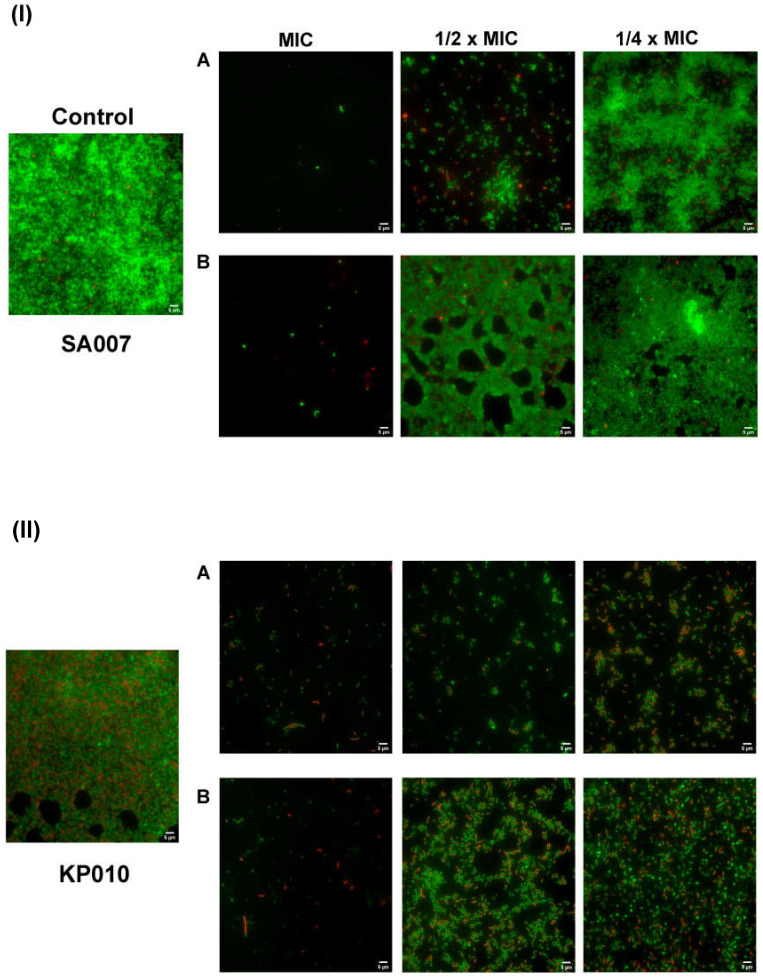
Fluorescence microscopy representative images of biofilms formed by MDR clinical isolates (**I**) *S. aureus* SA007, and (**II**) *K. pneumoniae* KP010, in the absence (control) or in presence of the test peptides (**A**) PP4-3.1 and (**B**) MeIm-PP4-3.1, at MIC, ½ × MIC and ¼ × MIC. The Live/Dead™ BacLight™ Bacterial Viability staining kit was used, which stains in green (SYTO 9 fluorescence) live bacteria, and stains in red (propidium iodide) dead or dying bacteria (with a damaged membrane). Scale bar corresponds to 5 µm.

The impact of PP4-3.1 and MeIm-PP4-3.1 on bacterial biofilm development was also assessed through a Live/Dead™ BacLight™ Bacterial Viability staining assay with subsequent observation by fluorescence microscopy. The same clinical isolates, SA007 and KP010, were used, and results are depicted in Figure 2. As expected, almost no bacteria could be observed at the MIC, i.e., biofilm formation was completely impaired. At sub-inhibitory concentrations, live bacteria (green) could be seen, and biofilm formation occurred to different extents depending on test peptide and bacterial isolate. Hence, it is quite clear that peptide PP4-3.1 (Figure 2(IA,IIA)) had a much stronger inhibitory effect on biofilm formation for both bacterial isolates, compared to the MeIm-PP4-3.1 analogue (Figure 2(IB,IIB)). These observations agree with data from the crystal violet assay, while offering string evidence of the superior performance of PP4-3.1, which becomes more obvious when comparing the images obtained at ½ × MIC.

### 3.4. Toxicity to Human Keratinocytes

The cytotoxicity of the test peptide constructs was assessed using immortalized human keratinocytes (HaCaT cell line), and the AlamarBlue™ assay for quantitation of metabolically active cells. The results are expressed as concentration of peptide causing 50% growth inhibition of the cells tested (IC_50_). As shown on Table 4, PP4-3.1 and the noncovalent equimolar mixture PP4:3.1 had similar IC_50_ values, which means that insertion of a covalent link between both building blocks was not disadvantageous regarding cytotoxicity. On the other hand, analogue MeIm-PP4-3.1 was almost twice more cytotoxic than its parent hybrid peptide PP4-3.1. Hence, introduction of the methyl imidazolium moiety was harmful towards the human cell line tested. This assay further revealed that peptide PP4-3.1 was not toxic for HaCaT cells at any of the MIC values obtained for all bacterial strains tested (see Figure S9, Supplementary materials), which highlights its higher selectivity.

### 3.5. Antifungal Activity

It is now well established that most cSSTIs have polymicrobial etiology, also involving fungal and bacterial pathogens. Indeed, although fungal colonization of non-healing wounds has been often overlooked [29], a high prevalence of fungal communities in chronic wounds has been previously reported and associated with both healing time and formation of mixed biofilms with bacteria [30]. Common fungal pathogens in cSSTI include *Cladosporidium* spp. and mainly, *Candida* spp., with *C. albicans* and *C. parapsilosis* as the most prevalent [31]. In view of this, we assessed the antifungal efficacy of PP4-3.1, MeIm-PP4-3.1 and the noncovalent equimolar mixture of PP4 and 3.1, on *C. albicans* (ATCC 90028), *C. glabrata* (ATCC 90030), and *C. parapsilosis* (ATCC 22019). The MIC values were determined according to the EUCAST protocol and are shown on Table 5.

In line with previous observations for antibacterial and antibiofilm activity, PP4-3.1 was once again the most potent peptide against all tested fungal strains, being even more active at lower concentrations than the reference antifungal drug, fluconazole, against *C. glabrata* and *C. parapsilosis*. The noncovalent mixture PP4:3.1 showed similar MIC values, but they were still higher than those of the covalent hybrid construct. Interestingly, the imidazolium derivative MeIm-PP4-3.1, though less potent than the PP4-3.1 peptide, was equally more active than fluconazole. In short, peptide PP4-3.1 further displayed a remarkable antifungal profile, with potent activity against all the *Candida* species tested, whose growing prevalence in nosocomial infections is a big concern [33].

### 3.6. Antibacterial Activity in Simulated Wound Fluid

Considering the relevant antibacterial, antibiofilm, and antifungal properties of peptide PP4-3.1, we further assessed its activity against *S. aureus* (ATCC 29213) in simulated wound fluid (SWF) to check if it remained active in a medium that more closely reflects a real wound. The SWF was prepared as previously reported [34], and *S. aureus* bacteria were allowed to grow in this medium in the presence of varying concentrations of peptide PP4-3.1. The MIC of PP4-3.1 in MHB was determined in the same assay, under identical conditions, for an accurate comparison. Results shown in Table 6 indicate an improvement of the antibacterial activity of PP4-3.1 in SWF, with MIC values against *S. aureus* as low a 0.3–0.5 µM. MBC values were identical to (MHB) or only slightly higher than (SWF) MIC values, ascribing a bactericidal action for this hybrid peptide.

## 4. Concluding Remarks

Antimicrobial peptides or, in a broader sense, host defense peptides (HDP), are frontrunners in providing new alternatives to current antibiotics, which are becoming virtually useless against the growing menace of multidrug resistant pathogens. While recent research has unveiled very promising HDP from either natural sources [35,36,37] or recombinant technology [38,39], most of the HDP that have been advanced over the past few years as prospective relevant players in the combat against infectious diseases are of synthetic origin. Rational design, along with chemical synthesis, offers a multiplicity of peptide-based constructs with no match in Nature, enabling a fine tuning of their pharmacodynamic and pharmacokinetic properties, thereby improving their fitness to reach the clinics [40,41,42]. *De novo* design has been delivering very promising HDP in the past couple of years such as, e.g., short amphiphilic peptides possessing the general structure K_n_F_m_K_n_ [43] and activity against antibiotic-susceptible strains of *E. coli* and *S. aureus* (2 ≤ MIC ≤ 32 mg/mL), or a structure based on IIKK repeat units conferring selective activity against antibiotic-susceptible *E. coli* (8 ≤ MIC ≤ 16 mg/mL) due to the ability to specifically interact with the outer membrane of this bacterium [44]. Computer-aided rational design of HDP is widening the chemical space around antimicrobial peptides [45], which, together with growing knowledge on specific features of bacterial cell surfaces, allows the tailoring of biomimetic peptides able to, e.g., overcome the extracellular polysaccharide capsule of *Klebsiella pneumoniae* [46] or specifically interact with the mycolic acid-rich envelope of *Mycobacterium tuberculosis*, killing this bacterium [47].

Our previous and current work provide further support to the value of novel synthetic peptide-based constructs by addressing the production of chimeric structures where different peptide and nonpeptide building blocks having distinct intrinsic biological properties, i.e., antimicrobial and collagenesis-inducing, have been combined in different ways to afford new improved constructs. In this connection, we have previously reported hybrid peptide 3.1-PP4, encompassing an *N*-terminal antimicrobial peptide sequence (3.1) directly linked to a well-known collagenesis-boosting peptide (PP4) as the *C*-terminal segment. This hybrid peptide stood out as a very promising lead for the development of topical agents for efficient management of skin infections, since it combines collagenesis-inducing ability with a potent action against Gram-negative bacteria, including MDR clinical isolates in either planktonic form or when forming biofilm structures. Moved by the clinical relevance of Gram-negative resistant bacteria, and the current lack of clinical options to fight them, we further explored the effect of adding an *N*-terminal modification (insertion of a methyl imidazolium group via “click” chemistry) to this peptide, which resulted in retained antibacterial activity and improved resistance to tyrosinase-mediated modification. However, the 3.1-PP4 construct had an Achilles’ heel, namely, its somewhat modest activity against Gram-positive bacteria, including *S. aureus*, which has great clinical relevance. Most skin and skin structure infections have a polymicrobial etiology, encompassing not only Gram-negative species but also Gram-positive bacteria and fungi. Indeed, while MRSA remains one of the major culprits for the chronicity and severity of cSSTI, involvement of fungal pathogens, especially *Candida* spp., is frequent and directly influences infection progress and wound healing. Having this in mind, and based on our previous findings, we explored the antibacterial, antibiofilm, and antifungal properties of the peptide construct PP4-3.1, as well as of its methyl-imidazolium derivative (MeIm-PP4-3.1) and of a noncovalent 1:1 mixture of its peptide building blocks (PP4:3.1). Altogether, results obtained are remarkable, as they allow us to advance peptide PP4-3.1 as a potent wide-spectrum antimicrobial agent, including against MDR bacterial isolates of both Gram-positive and Gram-negative bacteria, with MIC values lower than most of the recently reported for other promising peptide-based antimicrobials, and also against *Candida* spp. Findings made also highlight how a simple switch in the order through which both parent peptides, PP4 and 3.1, are linked to each other may act as a “game changer”. Interestingly, while this demonstrates that apparently innocuous structural changes have significant impacts on the antimicrobial properties of peptide-based constructs, the other side of the coin is equally relevant in that specific features of bacterial strains may underpin the antimicrobial effects of HDP [48]. This emphasizes the need to explore, in depth, the modes of action of HDP, and how these can be influenced by peptide and pathogen-related specificities. As such, ongoing and future studies will provide a more accurate and complete profiling of PP4-3.1, encompassing (i) determination of IC_50_ values from growth curves of peptide-treated microbes in both medium and SWF, which will in turn allow for accurate calculation of selectivity indices; (ii) evaluation of peptide’s action on dormant/slow-growing bacteria, and (iii) investigation of putative mode(s) of action against different microbial species and strains, with focus on both microbial membrane permeabilization and immunomodulatory effects, which are commonly ascribed to HDPs [49].

To conclude, peptide PP4-3.1 is indicated as a relevant lead for topical use against cSSTI, which combines an efficient action in vitro against MDR clinical isolates of both Gram-positive and Gram-negative bacteria, including installation of their respective biofilms, with an antifungal activity that matches, or even outperforms that of fluconazole. Considering the polymicrobial nature of cSSTI and the described beneficial effect of adding fluconazole to the standard care of diabetic foot ulcers [50], our report paves the way towards future options in the post-antibiotic era.

## Figures and Tables

**Table 1:** General data on the peptide constructs synthesized.

Peptide ^a^	Sequence ^b^/Structure	MW/Da
PP4-3.1	KTTKSKKLLKWLLKLL	1940.5
MeIm-PP4-3.1	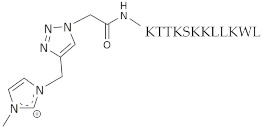	2144.8
PP4	KTTKS	562.7
3.1	KKLLKWLLKLL	1394.9

^a^ all peptides were produced as *C*-terminal amides; ^b^ amino acids are represented in single letter code as defined by the IUPAC-IUBMB guidelines on nomenclature and symbolism for amino acids and peptides.

**Table 2:** Activity of the peptide constructs against susceptible and MDR Gram-positive and Gram-negative bacteria (planktonic).

	Bacterial Species	Reference Strain or MDR Isolate		MIC in µM (in µg/mL) ^a^				
			CIP	PP4-3.1	MeIm-PP4.3.1	PP4	3.1	PP4:3.1 (1:1)
*Gram-negative*	*E. coli*	ATCC 25922	0.012 (0.004)	1.4 (2.8)	0.7 (1.5)	>60	3.8	1.9
	*P. aeruginosa*	ATCC 27853	0.18 (0.06)	1.4 (2.8)	1.4 (2.9)	>60	3.8	7.5
		PA004	96 (32)	1.4 (2.8)	1.4 (2.9)	ND ^b^	ND ^b^	ND ^b^
	*K. pneumoniae*	ATCC 13883	0.75 (0.25)	1.4 (2.8)	1.4 (2.9)	>60	3.8	7.5
		KP010	48 (16)	2.9 (5.6)	2.8 (5.9)	ND ^b^	ND ^b^	ND ^b^
*Gram-Positive*	*S. aureus*	ATCC 29213	1.5 (0.5)	2.9 (5.6)	2.8 (5.9)	>60	1.9	1.9
		SA007	193 (64)	1.4 (2.8)	1.4 (2.9)	ND ^b^	ND ^b^	ND ^b^
	*E. faecalis*	ATCC 29212	0.38 (0.125)	5.7 (11)	5.5 (12)	>60	3.8	1.9
	*S. epidermidis*	ATCC 14990	0.75 (0.25)	0.8 (1.5)	0.7 (1.5)	ND ^b^	ND ^b^	ND ^b^
	*S. pyogenes*	ATCC 19615	6.04 (2)	2.7 (11)	5.5 (12)	ND ^b^	ND ^b^	ND ^b^

^a^ MBC values were found to match MIC values in all cases; ^b^ ND, not determined. CIP: ciprofloxacin.

**Table 3:** Activity of the peptides against planktonic *S. aureus* and *K. pneumoniae* MDR clinical isolates in TSB.

Peptides	MIC in µM (µg/mL)	
	*S. aureus* (SA007)	*K. pneumoniae* (KP010)
PP4-3.1	1.3 (2.8)	5.7 (11.1)
MeIm-PP4-3.1	1.4 (2.9)	10.9 (23.4)
PP4:3.1 (1:1)	ND ^a^	15.0

^a^ ND, not determined.

**Table 4:** Toxicity of test peptide constructs to HaCaT cells, after 24 h of incubation.

Peptide	IC_50_ (µM) ^a^
PP4-3.1	13.0 ± 1.0
MeIm-PP4-3.1	5.7 ± 1.0
PP4:3.1 (1:1)	11.7 ± 1.1

^a^ results are expressed as mean ± standard error of the mean (SEM) of two independent experiments (*n* = 8).

**Table 5:** Activity of the peptide constructs, respective parent peptides, and reference antifungal drug fluconazole, against ATCC *Candida* spp.

Peptide	MIC in µM (µg/mL)		
	*Candida albicans* ATCC 90028	*Candida glabrata* ATCC 90030	*Candida parapsilosis* ATCC 22019
PP4-3.1	2.9 (5.6)	11.5 (22.2)	1.4 (2.8)
MeIm-PP4-3.1	10.5 (23.4)	43.7 (93.7)	2.8 (5.9)
PP4	>60	>60	>60
3.1	7.5	15	3.75
PP4:3.1 (1:1)	7.5	15	3.75
Fluconazole	1.6 (0.5)	52 (16)	6.5 (2)

**Table 6:** MIC and MBC values in µM (µg/mL) of PP4-3.1 against *S. aureus* (ATCC 29213) in MHB and SWF ^a^.

Peptide	MHB		SWF	
	MIC	MBC	MIC	MBC
PP4-3.1	2.1 (4)	2.1 (4)	0.3–0.5 (0.5–1)	1–2.1 (2–4)

^a^ results from three independent experiments performed in triplicates.

## Supplementary Materials

The following are available online at https://www.mdpi.com/article/10.3390/pharmaceutics13111962/s1. Figure S1: RP-HPLC chromatogram obtained for PP4-3.1. Figure S2: Full ESI-IT MS (positive mode) obtained for PP4-3.1. Figure S3: RP-HPLC chromatogram obtained for MeIm-PP4-3.1. Figure S4: Full ESI-IT MS (positive mode) obtained for MeIm-PP4-3.1. Figure S5: RP-HPLC chromatogram obtained for PP4. Figure S6: Full ESI-IT MS (positive mode) obtained for PP4. Figure S7: RP-HPLC chromatogram obtained for 3.1. Figure S8: Full ESI-IT MS (positive mode) obtained for 3.1. Table S1: Antibiotic resistance pattern of MDR clinical isolates PA004, KP010 and SA007. Figure S9: Cell viability (%) versus the logarithm of test peptide concentration (µM). Figure S10: Supplementary fluorescence microscopy images representative of biofilms formed by MDR clinical isolates (I) *S. aureus* SA007, and (II) *K. pneumonia* KP010, in absence (control) or in presence of the test peptides (A) PP4-3.1 and (B) MeIm-PP4-3.1, at MIC, ½ × MIC and ¼ × MIC.
