# Supplementary material for: Disclosure of a Promising Lead to Tackle Complicated Skin and Skin Structure Infections: Antimicrobial and Antibiofilm Actions of Peptide PP4-3.1
Source: Pharmaceutics. 2021 Nov 19;13(11):1962. doi: 10.3390/pharmaceutics13111962 (PMC8619843; doi:10.3390/pharmaceutics13111962)
Supplement: Supplementary file 1 [file pharmaceutics-13-01962-s001.zip › pharmaceutics-1409000-SI.pdf]

pharmaceutics

MDPI

Supplementary Materials: Disclosure of a Promising Lead to Tackle Complicated Skin and Skin Structure Infections: Antimicrobial and Antibiofilm Actions of Peptide PP4-3.1

Ana Gomes, Lucinda J. Bessa, Iva Fernandes, Ricardo Ferraz, Cláudia Monteiro, Cristina Martins, Nuno Mateus, Paula Gameiro, Cátia Teixeira and Paula Gomes

INSTRUMENTAL ANALYSIS

All RP-HPLC analyses were performed in a Hitachi-Merck LaChrom Elite system equipped with an L-2130 quaternary pump, an L220 thermostatted automated sampler, and an L-2455 diode-array detector (DAD). The samples were injected in a reverse-phase C18 column (125×4.0 mm ID and 5 μm pore size) with the elution gradient of 1 to 100% of B in A, using 0.05% aqueous trifluoro acetic acid (TFA) as solvent A and acetonitrile (ACN) as solvent B, and was run for 30 min at the flow rate of 1mL/min. The detection was performed at 220 nm.

All ESI-IT-MS analyses were performed in a Finnigan Surveyor LCQ DECA XP MAX spectrometer operating with electrospray ionization and ion trap quadrupole detection, at the department of Chemistry and Biochemistry University of Porto.

Chromatographic and spectral traces thus obtained are given below.

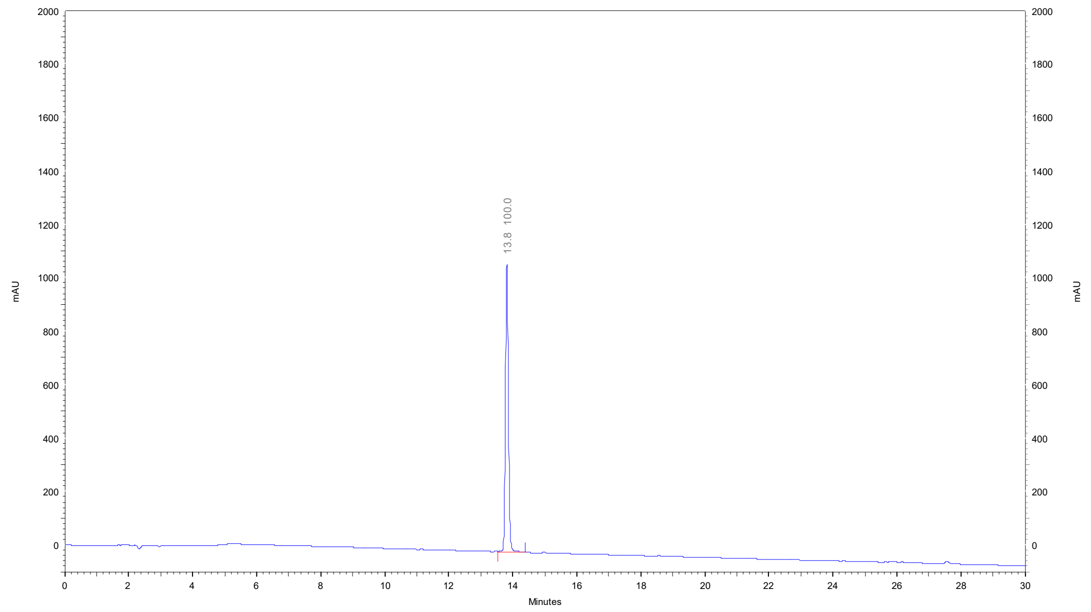

Figure S1. RP-HPLC chromatogram obtained for PP4-3.1.

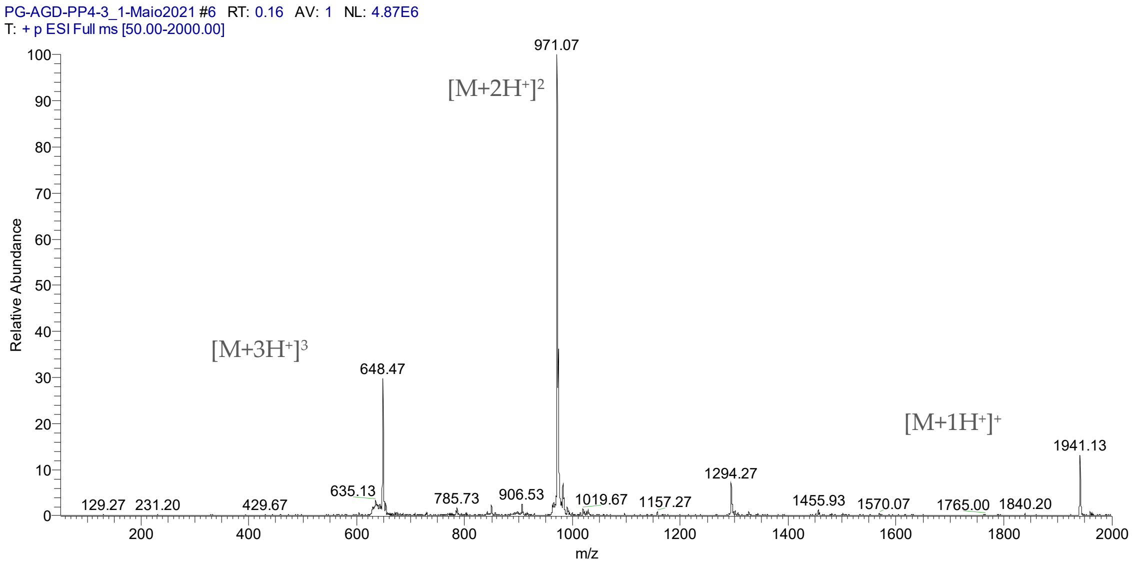

Figure S2. Full ESI-IT MS (positive mode) obtained for PP4-3.1.

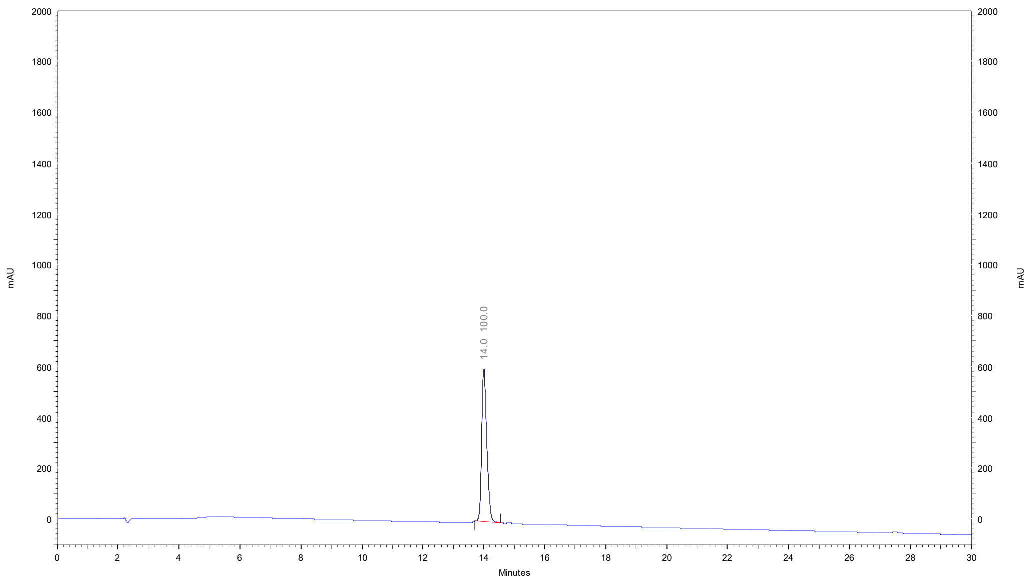

Figure S3. RP-HPLC chromatogram obtained for MeIm-PP4-3.1.

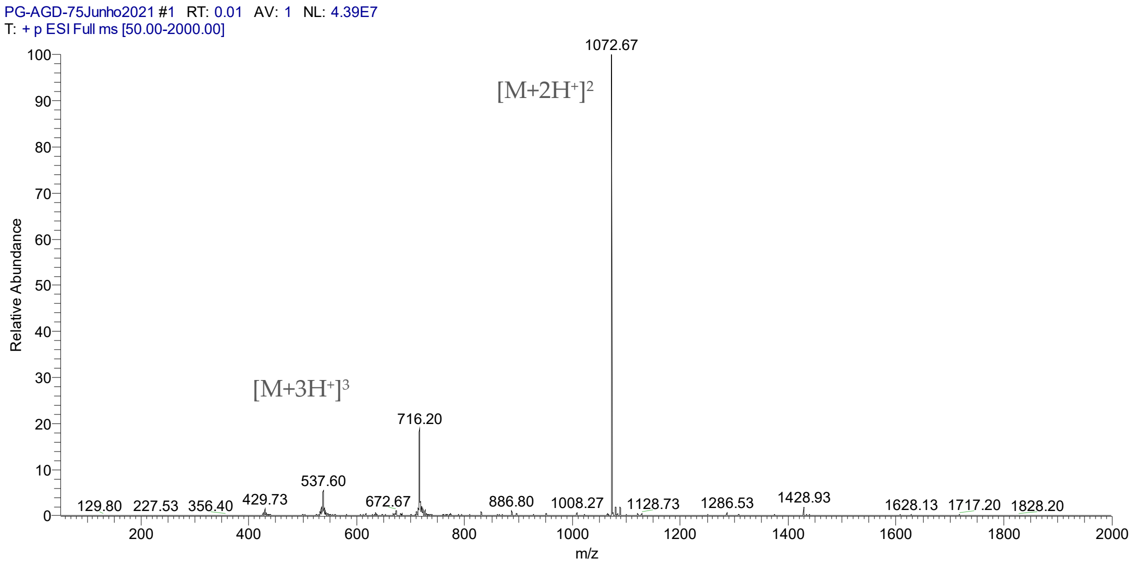

Figure S4. Full ESI-IT MS (positive mode) obtained for MeIm-PP4-3.1.

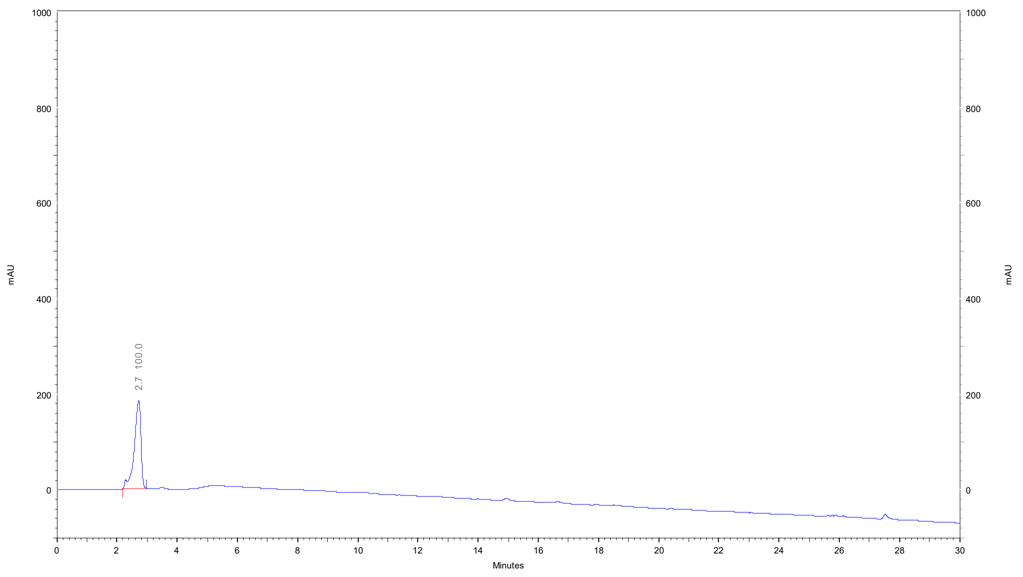

Figure S5. RP-HPLC chromatogram obtained for PP4.

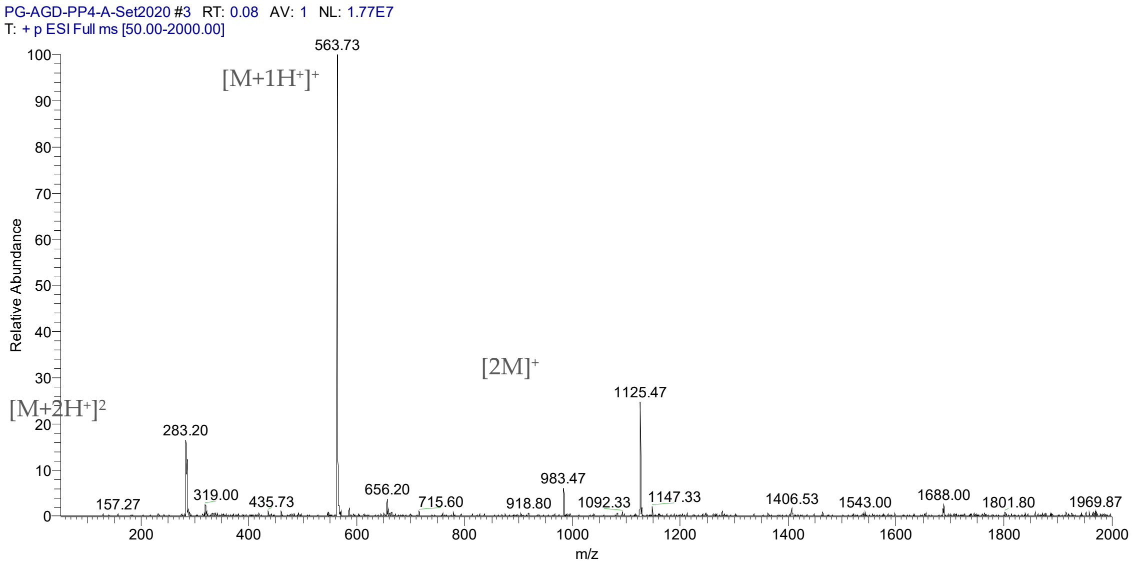

Figure S6. Full ESI-IT MS (positive mode) obtained for PP4.

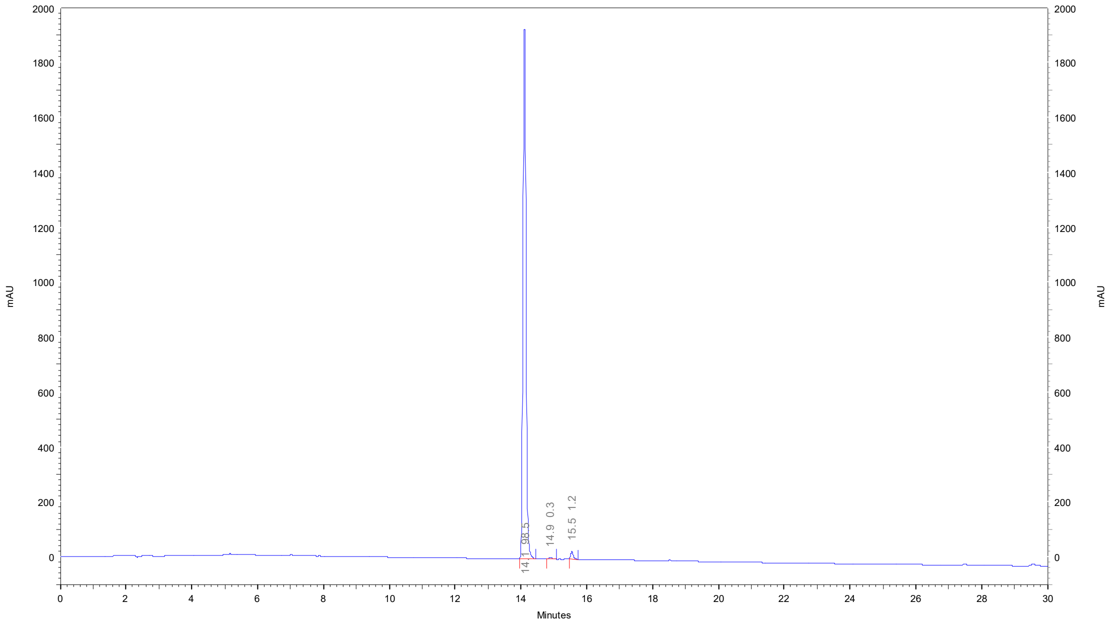

Figure S7. RP-HPLC chromatogram obtained for 3.1.

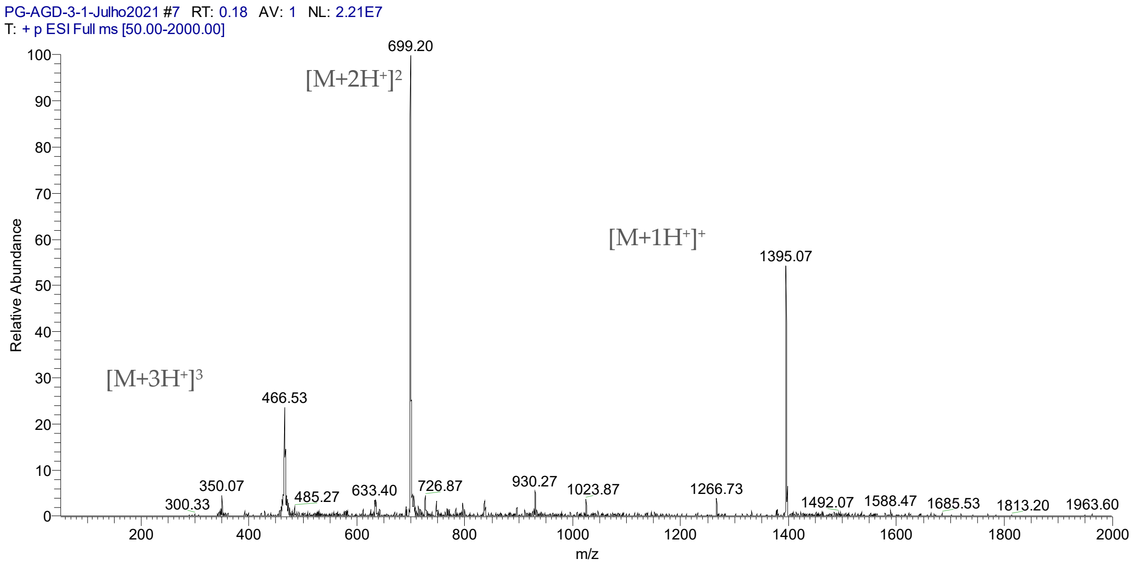

Figure S8. Full ESI-IT MS (positive mode) obtained for 3.1.

Table S1. Antibiotic resistance pattern of MDR clinical isolates PA004, KP010 and SA007.

Isolate	Antibiotic resistance pattern
PA004	CIP, GEN, IPM, TOB, TZP
KP010	AMC, AMP, CAZ, CIP, CTX, CXM, ERT, IPM, LEV, NIT, TZP
SA007*	CIP, CLI, ERI, FOX, GEN, LEV, MOX, OXA

AMC: amoxicillin/clavulanic acid; AMP: ampicillin; CAZ: ceftazidime; CIP: ciprofloxacin; CLI: clindamycin; CTX: cefotaxime; CXM: cefuroxime sodium; ERI: erythromycin; ERT: ertapenem; FOX: cefoxitin; GEN: gentamicin; IPM: imipenem; LEV: levofloxacin; MOX: moxifloxacin; NIT: nitrofurantoin; OXA: oxacillin; TOB: tobramycin; TZP: piperacillin/tazobactam; * Methicillin-resistant *S. aureus* (MRSA)

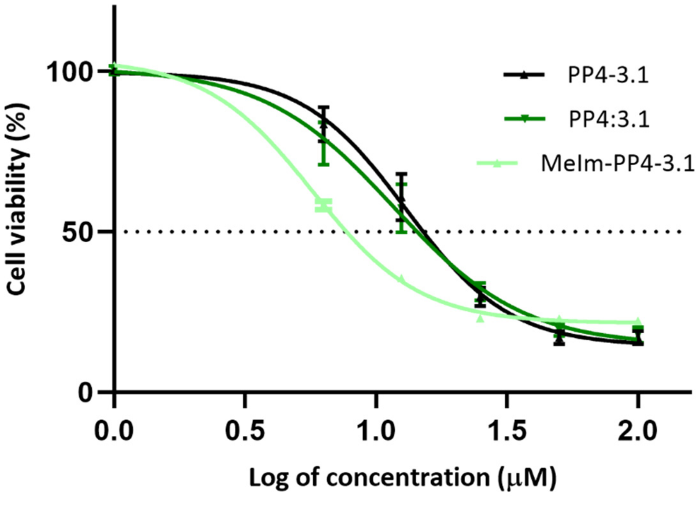

Figure S9. Cell viability (%) *versus* the logarithm of test peptide concentration (μM).

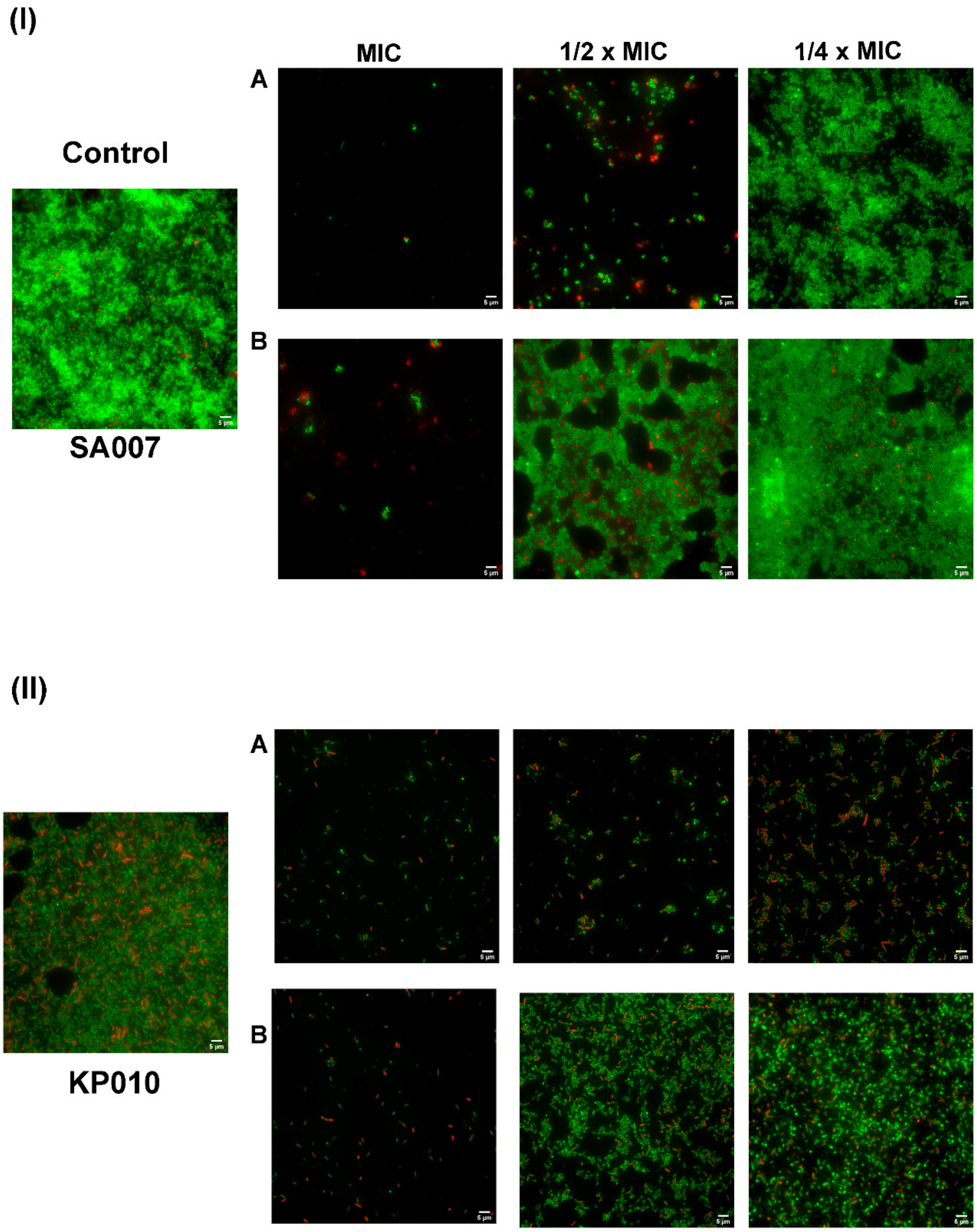

Figure S10. Supplementary fluorescence microscopy images representative of biofilms formed by MDR clinical isolates (I) *S. aureus* SA007, and (II) *K. pneumonia* KP010, in absence (control) or in presence of the test peptides (A) PP4-3.1 and (B) MeIm-PP4-3.1, at MIC, ½×MIC and ¼×MIC.
